# Diet in Disease

**Published:** 1893-03-25

**Authors:** Ernest Hart

**Affiliations:** Bachelier-ès-Sciences-es-Lettres (rèstreint), formerly Student of the Faculty of Medicine of Paris, and of the London School of Medicine for Women


### Diet in Disease.

By Mrs. Ernest Hart, Bachelier-ès-Sciences-es-Lettres (rèstreint), formerly Student of the Faculty of Medicine of Paris, and of the London School of Medicine for Women.

### XVII.—GOUT.

Gout seems to be the most ancient, the most persistent, and still the most incomprehensible of diseases. Its origin, its cause, and its cure are almost as little understood now as in the time of the Romans. Any number of views have been promulgated, but after reading them the poor student in search of knowledge is more confused than he was before, when he held the simple and popular opinion that chalk stones meant gout, and that colchicum relieved the attacks. Among many opinions that of Dr. Todd seems to be the most practical, when he says, "There is no disease in which the patient can do so much for himself, or in which the prescriptions of the physician are of so little avail without the full and complete co-operation of the patient, as in gout." So far as we understand gout it seems to be caused by a want of balance between the intake of food and the power of the body to oxidise and utilise it; and can be controlled by the patient checking the consumption of food, and taking means to promote oxidation. Let me explain. It will be remembered that when I described the part played by albumen in metabolism, I showed how albumen and the foods containing that substance were oxidised in the body. After yielding the nitrogen necessary for the reconstruction of the vital fluids of the body, the final product of albumen is urea, which is separated from the blood by the action of the kidneys, and being very soluble, is dissolved in the urine and cast out of the body as a waste product. Now, it can easily be understood that if by some fault in the organism the oxidation of albumen is not completed and carried on to the final production of urea, but stops short at the production of a less highly oxidised substance, namely, uric acid, there will be a disturbance of the ordinary course of action in the body. This uric acid is not, moreover, soluble like urea, and cannot be carried off by the kidneys with the same facility, being like an insoluble and intractable substance. It exists in the form of small pointed crystals, which cause irritation of the urinary passages; and, circulating in the blood, uric acid enters into combination with the sodium of the serum, and forms acicular crystals of urate of sodium, which are deposited in the membranes of the joints, giving rise to the well-known symptom of gout, namely, chalk stones. The attacks of gout are caused by an effort of nature to get rid of these deposits of a foreign substance in the joints; the joint becomes acutely inflamed, there is an increased flow of blood to the part, and urate of sodium is discharged into the blood current.

Now it will be understood that if by some inherited or acquired vice of the constitution the gouty person has not the power of oxidising the amount of food ordinarily taken, there will consequently be a want of balance between the consumption of food and the elimination of waste products, with the inevitable result that a quantity of effete and injurious material remains circulating in the blood. The person threatened with gout must, if he would be healthy and wise, ascertain by careful observation and experiment the exact amount of food which his body has the power of oxidising, or he should by increased exercise and fresh air so stimulate oxidation in the body that the balance can be restored and maintained. There is no doubt that this can be done, but the gouty person must, if he would be free from attacks of his malady, become an ascetic in the matter of eating and drinking.

**The Intake of Food should be Strictly Limited.**—The gouty person should never eat to satiety, but only enough to maintain the strength and to restore the waste of the body. It is difficult to lay down any hard-and-fast rule on this question of the amount of food to be taken, as it would depend in a great measure on the amount of exercise taken daily. It is therefore a point that the patient must decide for himself after careful observation. I am acquainted with a vigorous old gentleman of 87, who successfully keeps the attacks of gout at bay, to which he is liable, by a frugal and scientific dietary. He breakfasts on fruit; at mid-day he takes a small meat meal consisting of 4 oz. of meat if he has walked twelve miles in the previous twenty-four hours, and of 2 oz. of meat if he has only walked six miles. His evening meal consists of baked apples, custard pudding, or some similar light dish. On this simple dietary, combined with active exercise, this octogenarian is able to live a busy public life, to be alert in mind and vigorous in body, and to ward off the gout, which would have killed a more self-indulgent person.

**The Kinds of Food to be Taken and Avoided.**—Foods containing starch and sugar should be avoided or taken in moderation. As already explained, starch is turned into glucose or sugar by the process of digestion, so that starch and sugar may be considered to have the same effect. Why sugar should have so deleterious an effect on the gouty is ill understood; but it is the common experience of gouty patients that sugar is poisonous to them. It is supposed that as starch and sugar are more easily and rapidly oxidised in the body than is albumen, and as in the gouty the power of oxidation of foods is impaired, if the usual mixed diet of foods containing albumen, starch, and sugar be taken, the body will seize on the more easily oxidisable starch and sugar, while the albumen will remain partly oxidised, thus causing the production of urate of sodium in the blood, which, as I have shown, is the cause of gout. For the same reason fatty foods should be taken in moderation, and also because they rapidly undergo acid fermentation in the stomach, and become, in some measure, the cause of the "acidity," so much complained of by gouty subjects. The diet should be limited to beef, mutton, chicken, game, fish, eggs, green vegetables, a few ounces of stale bread, and a small quantity of butter (Roose). Fruit may be allowed, if not too sweet, and if found by experience not to disagree. Tea and coffee should be used in moderation. Cocoa made from nibs is recommended. Milk is ill-tolerated by some, but in other cases from one to two pints a day can be taken with advantage. Pastry of all kinds is forbidden. Bread, rice, potatoes, beans, and peas, all of which contain a large amount of starch, should be taken only in small quantities. The gouty person should not therefore be a vegetarian, for the vegetarian in order to obtain the amount of albumen necessary for tissue change in the body is obliged to take with it a large amount of starch, as vegetable albumen is contained in beans and peas, which are full of starch. The gouty should, however, be a total abstainer, for it is indisputable that alcohol in any form is injurious. Excessive beer drinking is often the cause of gout among the poor, while a long course for many generations of "high living" is the fruitful source of gout among the well-to-do. If the enfeebled digestive powers need the stimulus of alcohol, old brandy or whiskey, well diluted, or good claret or hock, are the most suitable and least injurious drinks. Water is the only article of diet the gouty may take in excess. It is well for him to drink an abundance of water, either hot or cold as he may prefer. Water washes out the tissues, augments secretion, and by removing waste products may prevent deposits of uric acid and urate of sodium. The various effervescing alkaline waters may often be substituted with advantage for plain water, and as lime juice is recommended to those who are unable to take vegetables, a lemonade made of an effervescing alkaline water with lime-juice and saccharin will be found a most refreshing and agreeable drink.

**Exercise and Fresh Air.**—It is important not only to limit the amount of food to be oxidised and disposed of in the body, but to increase the power of oxidation. This is best done by exercise and fresh air. Exercise stimulates the circulation, promotes tissue change, and increases oxidation in the body; hence it is obvious that the maintenance of that delicate balance between the food taken and the oxidation of that food, which is so necessary for the gouty, can be greatly aided by exercise. The exercise must not, however, be too violent and fatiguing, for anything that tends to depress the nervous powers may cause an attack of gout. Undue excitement, sleeplessness, over study, anxiety, should, therefore, all be avoided by the gouty. If, owing to stiffness of the joints, active walking or horse exercise cannot be taken, passive exercise should be resorted to, such as can be obtained by means of massage. Fresh air is of great importance, though damp and cold should be avoided. Great benefit may be obtained by a sojourn in a warm dry climate, such as that of Las Palmas in Grand Canary or Oratavo in Teneriffe. Fine hotels and the best medical attendance can now be found at these health resorts, and it will, I think, not be long before the martyrs to arthritic gout will learn that the "Fortunate Islands" may be to them a discovery worth making.

### *A WEEK'S MENUS.*

First Day.

*Breakfast.*

A boiled egg and dry toast.
Cocoa from nibs with added milk and sweetened with saccharine. (1)

*Lunch.*

Baked apples and custard pudding.
Lime-juice lemonade. (2)

*Dinner.*

Tomato soup.
Roast chicken and asparagus.

(1) The discovery of saccharine has been as valuable to the gouty as to the diabetic. It should be used in the place of sugar.

(2) This is an excellent drink. It is made with Apollinaris or soda water, to which is added one teaspoonful of lime-juice and a tabloid of saccharine to the pint.

Second Day.

*Breakfast.*

Grilled sole with lemon-juice and butter.
Tea and toast.

*Lunch.*

Stewed cabbage. (3)
Stilton cheese and rusks.

*Dinner.*

Bisful Soup.
Mutton cutlets and French beans.
Junket.

(3) Take a good-sized savoy, or spring cabbage; cut, wash, and use only the heart; boil it for a quarter of an hour. Meanwhile take 2 oz. of fat bacon chopped fine, a little onion and parsley, and a few herbs. Brown together in a stew-pan. Put in the boiled cabbage and stew altogether for about three-quarters of an hour.

Third Day.

*Breakfast.*

Eggs with black butter.
Fresh fruit.
Coffee and toast.

*Lunch.*

Artichoke soup. (4)
Sardines on toast.

*Dinner.*

Oysters.
Fillet of beef au printanière and potatoes.
Stewed rhubarb and cream.

(4) The various soups made of vegetable purées are excellent for gouty patients. They are sufficiently sustaining to prevent a feeling of hunger, and if well digested they give a fair amount of nourishment.

Fourth Day.

*Breakfast.*

Fried cutlets of cod.
Milk and soda water (7), toast.

*Lunch.*

Banana fritters.
"Cart-wheel" (8) and pulled bread and butter.

*Dinner.*

Spinach soup.
Roast pheasant and broccoli.
Almond pudding. (9)

(7) Milk is often found to be more digestible if diluted with an alkaline effervescing water. So treated, it makes also a more agreeable drink than when taken pure. Should the physician prescribe the avoidance of milk, cream and aerated water will be found to make a most nutritious and agreeable drink, particularly for breakfast, if tea and coffee cannot be taken.

(8) This is a cheese which goes by this name owing to its immense circular size. It is made of skim milk, and is thus free from fat. In cases when the richer cheeses cannot be well digested "cart-wheel" may form a useful food for the gouty.

(9) The recipe for this has already been given.

Fifth Day.

*Breakfast.*

Poached eggs on toast.
Tea.

*Lunch.*

Boiled sole.
Tomato salad.

*Dinner.*

Victoria soup.
Roast grouse.
Asparagus.

Sixth Day.

*Breakfast.*

Cold ham.
Melon.

*Lunch.*

Stewed plums and rice.

*Dinner.*

Boiled chicken with sautés potatoes.
Cauliflower au gratin.

Seventh Day.

*Breakfast.*

Finnan haddock.
Nib cocoa and milk, toast.

*Lunch.*

Cock à leekie soup.
Cheddar cheese and Callard's biscuits. (10)

*Dinner.*

Cutlets of salmon.
Roast mutton with
Potatoes and cauliflower.
Gooseberry fool.

(10) Callard's biscuits and cakes are as useful to the gouty as to the diabetic. They are carefully and intelligently made, are free from starch and sugar, but are yet very pleasant to eat.

